# Supportive Home Remedies for Orofacial Pain during the Coronavirus Disease 2019 Pandemic: Their Value and Limitations

**DOI:** 10.1155/2022/2005935

**Published:** 2022-01-20

**Authors:** Yeon-Hee Lee

**Affiliations:** Department of Orofacial Pain and Oral Medicine, Kyung Hee University Dental Hospital, #613 Hoegi-dong, Dongdaemun-gu, Seoul 02447, Republic of Korea

## Abstract

**Background:**

The coronavirus disease 2019 (COVID-19) pandemic has impeded access to timely dental care, and there is an urgent need for adjuvant therapies that can reduce orofacial pain in emergencies.

**Aims:**

To provide information on the benefits and limitations of eight representative home remedies as palliative care for orofacial pain during the coronavirus disease 2019 (COVID-19) pandemic.

**Methods:**

PubMed and Medline were electronically searched for eight home remedies for orofacial pain that can be used in COVID-19. Papers published in English in the past 30 years were considered. Among the published studies suitable for the research purpose, those in which the abstract and body text were confirmed were targeted, and duplicate studies were excluded. Finally, 86 studies were included.

**Results:**

There is extensive and high-level scientific evidence for the application of tooth brushing and flossing, mouth rinsing with chlorhexidine, use of over-the-counter pain medication, and application of cryotherapy in emergencies. Gargling with salt water, brushing with bamboo salt, gargling with garlic juice, and oil pulling are traditional methods used for centuries. The use of natural products for orofacial pain has a significant empirical effect but has weak scientific evidence.

**Conclusions:**

Knowing the correct application method, effects, and side effects is desirable to use these methods appropriately in emergencies. However, scientific evidence is unclear and generally lacking for home remedies to be the main treatment strategy, and there are clear limitations to their use as a single main treatment.

## 1. Introduction

During the coronavirus disease 2019 (COVID-19) pandemic, professional dental treatment has not been immediately available in many cases, such as self-quarantine and quarantine in medical facilities due to confirmed COVID-19 or close contact with an infected individual. Moreover, due to the COVID-19 pandemic, people have been avoiding dental treatment due to the fear and anxiety of infection with severe acute respiratory syndrome coronavirus 2 (SARS-CoV-2), the cause of COVID-19 [1]. COVID-19 continues to spread worldwide, threatening public health and the economy with nearly 250 million confirmed cases and more than five million deaths [2, 3]. Additional barriers to seeking timely dental care are considered stressful [4]. Recently, it has become more difficult to manage and treat orofacial pain than ever before. Therefore, routine dental care is occasionally delayed, and patients may address emergencies using alternatives that are not strongly recommended.

The International Classification of Oral and Facial Pain (ICOP), the first comprehensive classification uniquely addressing orofacial pain, was published in 2020 [5]. According to the ICOP, dental pain is defined as pain caused by lesions or disorders affecting one or more teeth and/or immediately surrounding and supporting structures—the tooth pulp, periodontium, and gingivae [5, 6]. Orofacial pain includes not only dental pain, but also pain caused by diseases, injuries, or abnormal functioning of the oral mucosa, salivary glands, jaw bone, or masticatory muscles. The best strategy of action for dealing with dental pain is to perform a diagnosis and administer proven treatment by a dentist [4]. In general, dental pain is usually unbearable and interferes with daytime activity and nighttime sleep. Due to COVID-19-related stress and insomnia, dental problems cause more orofacial pain or discomfort in the general population [7].

As oral health and general health are closely related, efforts to alleviate orofacial pain should not be neglected during the public health emergency of COVID-19 [8]. Active and appropriate approaches are needed to reduce dental pain [9]. To avoid terrible pain in the context of COVID-19, professionals and clinicians should provide information on appropriate home remedies, even if the remedies have an unscientific or weak scientific basis. The major cause of pain is the release of inflammatory mediators that activate sensory nociceptors surrounding the tooth [10]. Delayed and chronic pain exacerbates the patient's psychological aspects, such as depression and anxiety, and increases peripheral and central sensitization [11]. Thus, timely efforts should be made to ensure that dental pain itself does not persist. Pain relief is of paramount importance when treating dental patients as pain has far-reaching effects on both patients and clinicians.

**Figure 1:**
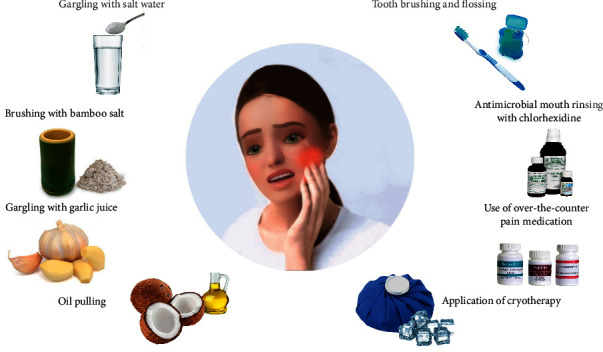
Eight representative methods can be used as alternative treatments for orofacial pain in the COVID-19 pandemic.

In this narrative review, we have summarized and presented the published scientific evidence on the action mechanisms, useful outcomes, risks, and limitations of eight representative methods used as alternative treatments (Figure 1). Narrative review articles are common in the medical literature and better address a wider topic than systematic review articles [12]. Home remedies are based on insufficient scientific evidence and have a low level of validation compared to the dentist's professional dental treatment protocols. However, rethinking and reconceptualization of dental care are required in a pandemic emergency, and home remedies are likely to play a sufficient role as palliative care for the main method.

## 2. Methods

### 2.1. Data Sources

In this narrative review, a comprehensive search of the literature was designed and performed using both natural language terms and controlled vocabulary for home remedies for orofacial pain under the COVID-19 pandemic situation. An electronic search was performed during September 2021 via PubMed, Medline (Ovid), Embase (Ovid), and Google Scholar. They were queried using the following keywords: “home remedy,” “natural product,” “dental caries,” “orofacial pain,” “dental pain,” “tooth pain,” “toothache,” “dental,” “dentistry,” “diagnosis,” “treatment,” “dental care,” “palliative care,” “oral health,” “management,” and “COVID-19” in combination. The published literature, including clinical research, original research, and literature review articles, was searched for home remedies for orofacial pain that can be used in COVID-19. Furthermore, other studies that evaluated physiological action mechanisms and treatment outcomes were reviewed. Studies published in English over the past 30 years were considered. Studies in which the abstract and body text were confirmed to be relevant to the review's aim were critically analyzed and summarized. Studies in which the main text was not written in English or the main text could not be found and those that did not fit the review's aim were excluded. Furthermore, duplicate studies were excluded. Finally, of the 191 studies that addressed our topic, 90 were included.

## 3. Results

Even without immediate professional dental treatment, dental pain can be relieved to some extent. Although only a dentist can definitively diagnose and treat the cause of dental pain, many home remedies are available. These methods are based on long-standing beliefs, but in some cases, the scientific basis is poor or there are side effects.

### 3.1. Oral Care with Tooth Brushing and Flossing

Through regular and correct tooth brushing and dental flossing, good oral hygiene plays a pivotal role in preventing tooth decay and oral inflammation. Regular tooth brushing is fundamental and powerful for relieving dental pain and maintaining good oral health. It is recommended to brush teeth twice a day—in the morning and before going to bed. This applies to both children and adults [13]. It helps to remove bacteria such as *Streptococcus mutans* and *Lactobacillus* spp. and plaque that cause tooth decay, periodontitis, and other oral diseases [14]. High-quality tooth brushing with gentle force while thoroughly cleaning the lingual and palatal sides rather than only the occlusal surface of the teeth is reasonable [13, 15].

However, brushing too hard or too frequently may cause gum pain, gingival recession, and tooth hypersensitivity. In addition, contaminated toothbrushes can play a role in microbial transport, growth, and retention [16]. Prolonged use of the toothbrush facilitates contamination by various microorganisms such as *Streptococcus*, *Staphylococcus*, *Lactobacillus*, *Pseudomonas*, *Klebsiella*, *Escherichia coli*, and *Candida* spp. [17]. These microorganisms are implicated in dental caries, gingivitis, stomatitis, and infective endocarditis in an individual. This can be the cause of reinfection in a person with pathogenic bacteria or a reservoir for harmful microorganisms. When the brush is trimmed, the end of the bristle has an irregularly shaped lumen, and fluids can be drawn into this core by capillary action, allowing for bacterial growth and contamination [18]. Therefore, it is necessary to change the toothbrush once every 3 months and use toothpaste with antibacterial action.

Dental flossing is the practice of flossing to remove food impaction that gets stuck into the interproximal space through occlusal pressure or plaque located along the gum line. Food impaction can increase the risk of oral and dental diseases such as halitosis, dental caries, gingivitis, periodontitis, and even tooth loss. In one cross-sectional study, flossing was associated with a modestly low prevalence of periodontitis [19]. Dental flossing helps eliminate the causes of toothache both physically and biologically. Use of floss or interdental brushes in addition to tooth brushing may reduce gingivitis, plaque, or both more compared to tooth brushing alone [20]. In a randomized trial, flossing was effective in reducing gingival bleeding and interproximal gingival inflammation [21]. As chronic gingivitis is initiated primarily due to biofilm accumulation, the standard practice of treatment is the removal of biofilm by mechanical disruption, such as tooth brushing and flossing [22]. However, misuse and overuse of floss can also increase gum inflammation and pain. Flossing 2–4 days a week could be as beneficial as flossing more frequently [19].

Furthermore, maintaining good oral health can be the best way to prevent the spread of SARS-CoV-2 and reduce related oral symptoms. The virus is mainly transmitted from human to human via droplet transmission and direct contact with oral, nasal, and eye mucous membranes [23]. Moreover, normal speaking also produces thousands of oral fluid droplets with a broad size distribution (1–500 *μ*m) [24]. In particular, salivary droplets and aerosols generated by infected and asymptomatic carriers are considered the disease transmission routes of SARS-CoV-2. High viral loads of SARS-CoV-2 have been detected in the saliva of COVID-19-positive patients, even in asymptomatic patients [25]. In one cross-sectional study, COVID-19-positive patients had signs and symptoms in the orofacial area, including headache, facial pain, masticatory muscle pain, dry mouth, burning mouth, taste disturbance, and oral ulcer [26]. In one clinical study, tooth brushing with water alone helped reduce SARS-CoV-2 [27]. As oral health is closely related to general health and managing oral hygiene can reduce the viral loads in the oral cavity, its importance in the COVID-19 pandemic deserves further emphasis.

### 3.2. Antimicrobial Mouth Rinsing with Chlorhexidine

Chlorhexidine gluconate is the best oral mouthwash to date. Chlorhexidine is a chemical antiseptic with a broad spectrum of bacteriostatic and bactericidal properties against both Gram-positive and Gram-negative microbes as well as antiviral and antifungal properties [28]. Regarding bacteriostatic and bactericidal properties, chlorohexidine damages the cytoplasmic membrane of microorganisms and disrupts the integrity of the cell membrane, causing leakage and precipitation of cytoplasmic protein and nucleic acids, thus destroying microorganisms [29]. Chlorhexidine can inhibit *Candida* adhesion to dental plaque; it acts as a fungicide, causing coagulation of nucleoproteins and changes in the cell wall, possibly leading to the escape of cytoplasmic components through the plasma membrane [28]. Although the mechanism is unclear, chlorohexidine also has antiviral effects, including against SARS-CoV-2 in the saliva [30].

Chlorhexidine is the most effective antiplaque agent. Overnight immersion of a toothbrush in chlorhexidine gluconate was highly effective in preventing microbial contamination. Moreover, studies have considered the immersion time and reported that immersing in chlorhexidine (0.12%) for 20 min and chlorhexidine (0.2%) was adequate to decontaminate toothbrushes [31]. Plaque and calculus are the main factors associated with gingival inflammation. Chlorhexidine (0.2%) mouth rinse significantly reduced plaque growth and gingival inflammation compared to a placebo mouth rinse in a previous study [32]. Chemical agents are incorporated into mouth rinses and dentifrices as adjunctive measures in supragingival plaque control [33]. However, there is insufficient evidence to support claims that chlorhexidine reduces the risk of gingivitis, periodontitis, or the rate of periodontitis progression. Chlorhexidine alone cannot completely resolve the origin of periodontal disease or toothache. In addition, frequent use of chlorhexidine can cause discoloration of the teeth and tongue, loss of taste, burning sensation in the oral mucosa, and subjective dryness of the oral cavity [34].

### 3.3. Use of Over-the-Counter Pain Medication

Nonsteroidal anti-inflammatory drugs (NSAIDs) are among the most widely prescribed analgesics for managing dental pain [35]. The effects, indications, and side effects of NSAIDs have been scientifically proven. NSAIDs have been approved by the US Food and Drug Administration for over-the-counter (OTC) analgesic use [36] and can be divided into three groups: salicylates (such as aspirin, salicylic acid, and diflunisal), propionic acid derivatives (such as ibuprofen (Advil), naproxen, and ketoprofen), and the para-aminophenol derivative acetaminophen (Tylenol). NSAID use must also be categorized based on whether the drugs are relatively low-dose over-the-counter oral products taken occasionally or whether they are higher-dose or parenteral NSAIDs [37]. Despite conflicting evidence for and against NSAIDs, it is unclear if the previous evidence would apply to all NSAIDs at all doses in all dosing regimens. In addition, if an individual is pregnant, breastfeeding, or has any other medical condition, consultations with a clinician are needed before using these OTC pain medications [38].

The analgesic effect of NSAIDs is primarily the result of their inactivation of cyclooxygenase, an enzyme that converts arachidonic acid into eicosanoids such as prostaglandins and leukotrienes [39]. Cyclooxygenase-dependent mechanisms involved in the antinociceptive action of NSAIDs are present at both peripheral and central sites [40]. Regarding the mechanism of action of NSAIDs, NSAIDs have hepatic side effects ranging from asymptomatic elevations in serum aminotransferase levels to liver failure and death [41]. Acetaminophen (paracetamol) has been proposed as an alternative to NSAIDs, but there are issues with liver toxicity at high doses. Acetaminophen decreases acute orofacial pain and is frequently used in patients with toothaches [42]. NSAIDs, in particular ibuprofen, may upregulate the entry point for the virus, angiotensin-converting enzyme 2 receptors, and increase susceptibility to the virus or worsen symptoms of existing disease [43]. The use of ibuprofen is controversial; therefore, it should be used with caution in patients with COVID-19.

The use of OTC medications is often effective, but caution is needed to prevent misuse and overuse. One prior study found that nonprescription analgesics are frequently overused in patients with orofacial pain [44]. However, frequent regular use of OTC pain medication was associated with an increased risk of hypertension, hepatic injury, and hepatic failure [35, 45]. It is meaningless to use painkillers to manage orofacial pain, which requires the use of antibiotics, antiviral agents, and other agents. In the case of intraoral swelling and pulpal- and periapical-related orofacial pain, urgent management with antibiotics is needed [46]. A definitive diagnosis of orofacial pain and appropriate drug use is necessary.

### 3.4. Application of Cryotherapy

Cryotherapy is the local or general use of low temperatures in medical therapy. Cryotherapy has analgesic effects and reduces local edema; therefore, this treatment is an option for patients with painful and inflammatory oral lesions [47, 48]. In a literature review, cryotherapy resulted in vasoconstriction, reduction of edema, and diminished pain perception [49]. Thus, the application of cold temperature effectively relieves orofacial pain and reduces swelling and inflammation by constricting the surrounding blood vessels. Clinically, ice packs are commonly used after dental procedures such as tooth extraction or dental implant surgery, and spray and stretch techniques are applied to the myofascial pain area.

An evaluation of the antiswelling and analgesic effects of different ice pack therapy durations on soft tissue injuries and patient discomfort revealed 10 min as the optimal ice pack therapy duration for individuals with soft tissue injuries [50]. To inhibit signs of inflammation and achieve beneficial results with cryotherapy, skin temperature, normally 33°C, needs to be reduced to 10°C–15°C within 10–20 min. The time interval for cold applications varied from 10 min to continuous hours. There seemed to be a consensus among clinicians that cryotherapy should be applied for 10–20 minutes, followed by a rest period [49]. However, no clinical trials have been conducted to determine the optimal interval of cold application (time on/off) or the extended duration of cryotherapy after surgical procedures to attain the best therapeutic benefits. In addition, cryotherapy has potential complications, including dyspigmentation, alopecia, depressed scars, and tissue distortion [51]. However, the appropriate application time according to the cause of pain or the rest time between applications has not been standardized, and studies on the side effects of long-term use have not been conducted.

### 3.5. Gargling with Saltwater

Although saltwater is believed to cleanse oral bacteria and reduce swelling, this is controversial. A simple saltwater rinse is used to rinse the mouth for 10–15 s and then spat out. The use of a saltwater mouthwash after extractions is a custom and practice in dental surgeries worldwide. It is believed that warm water with salt helps ease periodontitis pain. However, the evidence for this practice seems to be nonexistent. Salt may have a slight anti-inflammatory effect. In his time-honored study, Stephan showed that plaque on the tooth surface, when exposed to sucrose, accelerated acid production, resulting in a pH drop that could only be gradually restored to the baseline plaque pH [52]. Regarding the mechanism of action of salt, saltwater rinses can be useful for all-around mouth health because they temporarily alkalize the mouth or increase its pH levels, deterring the proliferation of bacteria.

Conversely, the old saying “rubbing salt into the wound” suggests that salt makes things worse and results in more pain. In periodontitis, neither the use of salt scrub techniques nor salt water rinses is of practical help [53]. In addition, excess salt has been implicated in osmotic-mediated activation/inhibition of apoptosis or necrosis processes [54]. The antibacterial effect is only temporary and can be harmful due to tooth and gingival abrasion in the long term. Additional research is needed on salt concentrations and usage that do not harm oral tissues. Since there is no scientific evidence that a saline mouth rinse is not significantly different from a light twice-daily gargle with saline in terms of efficacy or advantage [55], a twice-daily routine gargle with sterile saline is recommended. Salt can be an alleviation factor for dental pain in some cases, or it can be an aggravator; therefore, it is recommended to use analgesic drugs first.

### 3.6. Brushing with Bamboo Salt

Bamboo salt is a medical salt that originated in traditional medicine in Korea. 1,000 years ago, bamboo salt was made with sea salt in a case made of young bamboo. Both ends of the bamboo case are sealed using natural ocher, and the bamboo is baked 3–9 times at 1,000°C–1,500°C using a pine tree as the fuel [56]. Currently, bamboo salt is one of the most well-known traditional medical treatments, not only in Korea but also in many other Asian countries [57]. Bamboo salt is believed to be effective in suppressing the growth of microorganisms and sterilization and has various therapeutic effects on numerous pathological conditions, including inflammation, viral diseases, diabetes, and cancer and its metastasis [58]. Brushing with bamboo salt has been used in folk medicine to alleviate periodontal disease, toothache, and bad breath. The potassium, calcium, magnesium, and iron content in bamboo salts are higher than those in purified and solar salts. Additionally, bamboo salt that has been baked for longer periods contains more minerals than purified and solar salts [59]. Increased levels of these minerals in the salt are important for enhancing antioxidant and anti-inflammatory effects [60]. Regarding anticancer mechanisms of purple bamboo salt on oral cancer, induction of apoptosis by increasing the number of apoptotic bodies and regulating the expression levels of the apoptosis-related Bax and Bcl-2 mRNA and proteins has been suggested [61]. Bamboo salt also exhibits a higher reduction potential; this may be because this type of salt contains more hydroxyl (OH) ions than purified or solar salts [62]. Bamboo salt can be used as a remineralization agent in incipient enamel caries lesions [63]. In previous studies, when bamboo salt and sodium fluoride solution were applied together, the surface strength of teeth increased and some degree of remineralization might have occurred [64, 65]. However, it is difficult to say that these results are because of bamboo salt alone, and scientific data on the effects of this salt are lacking.

### 3.7. Gargling with Garlic Juice

In both the East and West, there is a myth that rinsing the mouth with garlic (*Allium sativum*) juice can help relieve dental pain. Garlic has also played an important role in Sumerian and ancient Egyptian medicine. There is some evidence that garlic was fed to the athletes during the earliest Olympics in Greece to increase stamina. Garlic has been used as a folk medicine worldwide to prevent and treat various diseases from ancient times to the present day. A method of placing crushed garlic into the cavity of the tooth or rinsing with garlic extract for approximately 3 min is also widely prevalent on online websites.

Garlic has been accepted as a powerful antibacterial agent. Garlic contains bioactive compounds, such as flavonoids, pyruvate, thiosulfate, cysteine, and diallyl sulfide, which are responsible for its antioxidant activity [66]. Garlic extract can inhibit the growth of both Gram-positive and Gram-negative bacteria. In particular, garlic extract has antibiotic effects against *S. mutans*, a Gram-positive, facultative anaerobic microorganism that is the main causative bacteria of tooth decay [67]. *S. mutans* is one of the primary etiological organisms in dental caries development [68].

Garlic cloves consist of sulfur-containing chemicals, such as allicin, alliin, and ajoene [69]. Allicin (allyl 2-propenethiosulfinate or diallyl thiosulfinate) is the principal bioactive compound present in the aqueous extract of garlic or raw garlic homogenate. When garlic cloves are cut or crushed, they release the enzyme alliinase, which converts alliin to allicin, which is responsible for its antibacterial activity [67]. When roasted garlic is placed on the toothache area, garlic allicin stimulates nerve cells to relieve toothache [70]. In an in vitro study, garlic juice was more effective against oral cariogenic bacteria (*S. mutans* and *L. acidophilus*) than chlorhexidine mouthwash; therefore, it can be recommended as a new type of mouthwash [71]. The use of garlic for treating dental pain has some limitations. The anticarcinogenicity of garlic extract can inhibit bacterial growth only when used at a high concentration [72]. Its antibacterial effect is less effective with increasing biofilm thickness in the oral cavity [73]. Therefore, mechanical bushings, flossing, and regular professional dental aids are essential. There are no harmful complications when using garlic; however, it has an unbearable taste and smell. Therefore, some people are reluctant to eat raw garlic because of its pungent taste and smell [74].

Garlic is considered effective for toothaches and dental caries because of its antibacterial and antiviral effects. Garlic derivatives have shown antimicrobial effects against periodontal and carious pathogens, including *S. mutans*, *S. sobrinus*, *Porphyromonas gingivalis*, *Actinomyces oris*, *Fusobacterium nucleatum*, and *Aggregatibacter actinomycetemcomitans* [75, 76]. The use of garlic to treat oral candidiasis and recurrent aphthous ulcers has also been successful without complications [76]. However, the mechanism of action is unclear. Further studies are needed to elucidate the pathophysiological mechanisms of action of garlic and its efficacy and safety in the management of dental pain.

### 3.8. Oil Pulling

Oil pulling is a traditional treatment that originated in ancient Ayurvedic medicine in India. Currently, oil pulling is used worldwide. Recently, various online website advocates have suggested that it can treat cavities, kill bacteria, eliminate bad breath, treat bleeding gums, prevent cavities, and even prevent heart disease. Oil pulling therapy is considered a simple and cost-effective method to improve and maintain good oral health with no strict precautions required to follow the regimen. Moreover, it does not require any specialized oil, and any household oil (such as sunflower or any other vegetable oil) can be used. The limited evidence suggests that oil pulling with coconut oil may improve oral health and dental hygiene [77]. Coconut oil has shown significant antifungal activity, comparable to that of ketoconazole and chlorhexidine [28]. A recent review article reported that oil pulling might be as effective as chlorhexidine mouthwash in reducing dental plaque [78]. If used properly, the oil-pulling method with tooth brushing can serve as an adjuvant to specialized dental care.

A possible mechanism of action is related to the antioxidative properties of the oil. The linoleic acid and oleic acid in sesame oil possess antioxidative properties that reduce lipid peroxidation by reducing free radical injury to oral tissues. Lauric acid, a major component of coconut oil, has antimicrobial activity against several microorganisms [79]. First, oil pulling has an inhibitory effect on *S. mutans*, a pioneering bacterium implicated in dental caries. In addition, it has antimicrobial activity against *Helicobacter pylori*, *S. aureus*, *E. vulneris*, *Enterobacter*, and *Candida* spp., including *C. glabrata, C. albicans*, *C. stellatoidea*, *C. parapsilosis, C. tropicalis*, and *C. krusei*, as well as antiviral activity against various viruses [79].

While there are many online reviews of people who have positively evaluated the effectiveness of oil pulling, scientific evidence is lacking. Moreover, there have been case reports of lipoid pneumonia associated with oil pulling or mineral oil aspiration [80, 81]. Currently, there is insufficient information about the benefits and potential harm of oil pulling to help us decide whether we should do it or whether it is better to prioritize brushing or dental treatment. More clinical data based on a scientific approach are needed to provide evidence of action mechanisms and possible adverse events for long-term use.

## 4. Discussion

On March 11, 2020, the World Health Organization declared the COVID-19 outbreak a global pandemic. Vaccines and therapeutic applications are being developed, but there is a long way to achieve herd immunity [82]. Traditionally, both patients and clinicians considered that the diagnosis and treatment of dental pain by a dentist in a dental clinic is the best treatment strategy. In the context of the COVID-19 pandemic, the demand for temporary management or minimally invasive treatments has increased [83]. With globalization, another pandemic can occur at any time, and the dental field must also be prepared for this change. In addition to the pandemic, technological advances, including telemedicine, big data, and artificial intelligence, have modified these traditional concepts [84, 85].

The eight most representative home remedies, including tooth brushing and flossing; use of OTC medicine; cryotherapy; and traditional remedies with salt, bamboo salt, garlic, and oil pulling, were selected from the information on how to cope with dental pain overflowing on Internet websites. Their scientific evidence, including the possible mechanism of action, benefits, utilization methods, expected complications, and limitations, was comprehensively investigated through published research. Knowing how to use self-care home remedies to help maintain oral health and reduce dental pain can be a good strategy for responding to this emergency.

Regular tooth brushing and dental flossing prevent pain due to inflammation of the gums or tooth decay and effectively control the proliferation of cariogenic bacteria represented by *S. mutans* [22, 86, 87]. OTC pain relievers or chlorohexidine gargling, which has antimicrobial and antiviral effects, can provide direct and useful aid in reducing dental pain [45, 88]. Application of cryotherapy to painful or swollen areas is helpful [49]. However, long-term, large-scale studies on the appropriate application time, application dose, side effects, indications, and contraindications of these treatments are lacking. Knowing and investigating the appropriate home remedies for dental pain may become more important during the COVID-19 pandemic for immediate pain relief.

Gargling or tooth brushing with salt is an accessible, cost-effective home remedy that has been used since ancient times in the West and East to reduce pain [55, 65]. Bamboo salt has also been used in the East for several centuries to treat various types of pain and diseases [65]. Oil pulling, which started in ancient India and has recently been used worldwide, also has antibacterial and anti-inflammatory effects [79]. The important weakness of these traditional home remedies is that, unlike Western medicine, their indications, contraindications, preparation method, action mechanisms, appropriate doses, beneficial effects, risks, and side effects have not been scientifically proven, and the application method has not been standardized or generalized. Their analgesic, anti-inflammatory, and antibacterial effects have been proven empirically or supported by studies with low scientific evidence.

Despite these weaknesses, on a larger public health scale, basic patient care protocols focus on and highlight the importance of simple, preventive, and behavioral modification strategies [89]. Recently, various alternative or traditional medicinal treatments have gained popularity because of their natural origins, cost-effectiveness, negligible side effects, safety, and improved patient compliance. With the development of dental materials and technologies, the future is near where patients themselves can cope with emergencies without professional dental care. Realistically, prior to the practical application of these materials and techniques, more research is required to accumulate stronger scientific evidence to assert the role of these adjuvant therapies in pain control. Consistent efforts are needed to obtain homogeneous results from randomized controlled trials or meta-analysis of randomized trials for the eight palliative treatments in the dental field.

## 5. Conclusions

Oral health acts as a gateway to general health. During the COVID-19 pandemic, maintaining good oral health and controlling harmful oral microorganisms can be mutually and significantly interrelated with overall health and well-being. In particular, many people who self-quarantine and are restricted by limited outdoor activities have been treated at home where professional dental services can be limited, but urgent dental aids can be touched. The scientific application of these adjuvant home remedies can help reduce orofacial pain and obtain psychological stability in emergencies during the COVID-19 pandemic. Home remedies can help improve oral health and reduce orofacial pain, which can positively affect oral and general health in the context of COVID-19. Of course, timely dental diagnosis and immediate treatment are the best strategies for dental pain. In addition, it is necessary to be aware of the limitation that home remedies alone cannot completely eradicate the cause of orofacial pain.

## Data Availability

The data that support the findings of this study are available on reasonable request from the author (Y.-H.L).
